# The midgut transcriptome of *Aedes aegypti* fed with saline or protein meals containing chikungunya virus reveals genes potentially involved in viral midgut escape

**DOI:** 10.1186/s12864-017-3775-6

**Published:** 2017-05-15

**Authors:** Shengzhang Dong, Susanta K. Behura, Alexander W. E. Franz

**Affiliations:** 1 Department of Veterinary Pathobiology, University of Missouri, Columbia, MO 65211 USA; 2 Department of Animal Sciences, University of Missouri, Columbia, MO 65211 USA

**Keywords:** *Aedes aegypti*, Chikungunya virus, RNA-Seq, Protein meal, Saline meal, Immunity- related gene, Midgut escape barrier, Collagen IV degradation, Basal lamina, Serine collagenase

## Abstract

**Background:**

The mosquito *Aedes aegypti* is the primary vector for medically important arthropod-borne viruses, including chikungunya virus (CHIKV). Following oral acquisition, an arbovirus has to persistently infect several organs in the mosquito before becoming transmissible to another vertebrate host. A major obstacle an arbovirus has to overcome during its infection cycle inside the mosquito is the midgut escape barrier, representing the exit mechanism arboviruses utilize when disseminating from the midgut. To understand the transcriptomic basis of midgut escape and to reveal genes involved in the process, we conducted a comparative transcriptomic analysis of midgut samples from mosquitoes which had received a saline meal (SM) or a protein meal (PM) (not) containing CHIKV.

**Results:**

CHIKV which was orally acquired by a mosquito along with a SM or PM productively infected the midgut epithelium and disseminated to secondary tissues. A total of 27 RNA-Seq libraries from midguts of mosquitoes that had received PM or SM (not) containing CHIKV at 1 and 2 days post-feeding were generated and sequenced. Fewer than 80 genes responded differentially to the presence of CHIKV in midguts of mosquitoes that had acquired the virus along with SM or PM. SM feeding induced differential expression (DE) of 479 genes at day 1 and 314 genes at day 2 when compared to midguts of sugarfed mosquitoes. By comparison, PM feeding induced 6029 DE genes at day 1 and 7368 genes at day 2. Twenty-three DE genes encoding trypsins, metalloproteinases, and serine-type endopeptidases were significantly upregulated in midguts of mosquitoes at day 1 following SM or PM ingestion. Two of these genes were *Ae. aegypti* late trypsin (*AeLT*) and serine collagenase 1 precursor (*AeSP1*). In vitro, recombinant AeLT showed strong matrix metalloproteinase activity whereas recombinant AeSP1 did not.

**Conclusions:**

By substituting a bloodmeal for SM, we identified midgut-expressed genes not involved in blood or protein digestion. These included genes coding for trypsins, metalloproteinases, and serine-type endopeptidases, which could be involved in facilitating midgut escape for arboviruses in *Ae. aegypti*. The presence of CHIKV in any of the ingested meals had relatively minor effects on the overall gene expression profiles in midguts.

**Electronic supplementary material:**

The online version of this article (doi:10.1186/s12864-017-3775-6) contains supplementary material, which is available to authorized users.

## Background

Diseases caused by mosquito-borne viruses are world-wide among the most challenging public health burdens in tropical countries. Current measures to control arthropod-borne viruses (arboviruses) and their mosquito vectors are insufficient due to the lack of sustainable vector control strategies and vaccines [1]. Control efforts to reduce the vectorial capacity of mosquitoes for arboviruses in the field rely mainly on insecticide applications, removal of mosquito breeding sites, use of repellents, door/window curtains, and bed nets [1, 2]. *Aedes aegypti* is the primary vector for the currently most prevalent arboviruses, which include dengue virus (DENV), chikungunya virus (CHIKV), Zika virus (ZIKV), and yellow fever virus (YFV). An important component of a mosquito's vectorial capacity is its vector competence for a particular virus. Vector competence refers to the mosquito's intrinsic ability, based on its genetic make-up, to acquire, maintain, and transmit an arbovirus. Innate immunity and tissue barriers in the mosquito are important determinants of its vector competence [3]. Following ingestion of a viremic bloodmeal from a vertebrate host, the midgut is the first organ that becomes infected by the virus. Following replication in the midgut, the virus then disseminates to secondary tissues including nerve tissue, hemocytes, fatbody, and the salivary glands. However, to establish a systemic and persistent infection, an arbovirus needs to overcome at least four tissue barriers in the mosquito. These barriers include midgut infection and escape barriers and salivary gland infection and escape barriers [4]. Once the latter has been overcome by the virus, the mosquito can transmit the virus to another vertebrate host during probing.

Recently, our research group began to investigate the nature of the midgut escape barrier for arboviruses in *Ae. aegypti* [4–6]. Our observations so far suggest that a virus such as CHIKV disseminates from the midgut to secondary tissue during bloodmeal digestion by crossing the basal lamina (BL) surrounding the midgut organ [5]. In insects, the BL is a layer of extracellular matrix consisting of collagen IV and laminin and synthesized during early development by hemocytes and the fat body [7]. Earlier studies showed that the pore size exclusion limit of the mosquito BL is only 9–12 nm [4, 8]. The authors concluded that the BL has to be remodeled/degraded to allow arbovirus virions (50–80 nm in diameter) to pass through. Indeed, our preliminary observations suggest that during bloodmeal digestion, the midgut BL is temporarily degraded as shown by a diminished abundance of collagen IV (S. Dong, V. Balaraman, A. Kantor, J. Lin, D. Grant, N. Held, A. Franz; unpublished information, manuscript under review). Furthermore, BL degradation coincides with the time window during which CHIKV is disseminating from the midgut.

Matrix metalloproteinases (MMPs) have been regarded as the principal proteinases to be involved in BL degradation/remodeling processes [6, 9–12]. In order to obtain a comprehensive expression profile of mosquito genes contributing to the midgut escape of arboviruses, we conducted an RNA-Seq experiment based on RNA samples from midguts of mosquitoes, which had received a saline meal (SM) or a protein meal (PM) containing/not containing CHIKV. Recently, bovine serum albumin (BSA) has been reported as an alternative protein meal for *Ae. aegypti* and *Ae. albopictus*, which is taken up by the mosquito midgut and upon its digestion facilitates female vitellogenesis and egg production [13–15]. Thus, we substituted blood for a PM (consisting of BSA) and a SM (consisting of PBS) to reduce potential "background noise" caused by genes responsive to blood digestion rather than to the process of viral dissemination from the midgut.

Genetic interactions between *Ae. aegypti* and arboviruses have been extensively investigated in whole-genome based transcriptome/proteome analyses to reveal the mechanisms of arbovirus infection and transmission [16–23]. As a result, numerous candidate genes have been shown to be regulated by replicating viruses and some of these genes appeared to have antiviral properties in *Ae. aegypti* [24–26]. For example, 20 upregulated and 15 downregulated genes with similar expression profiles were identified at day 1 of infection in the Rockefeller strain of *Ae. aegypti*, which had been challenged with three different flaviviruses, West Nile virus (WNV), DENV2, and YFV [22]. These genes exhibited diverse functions including ion binding, transport, metabolic processes, and peptidase activity. A specific outcome of this study was the identification of a pupal cuticle protein (AAEL011045), which was able to bind WNV envelope protein, leading to inhibition of infection in vitro and the prevention of lethal WNV encephalitis in mice. Bonizzoni and colleagues showed that DENV2 infection affected the expression of 397 genes in *Ae. aegypti* (strain: Chetumal), 132 of which exhibited significant differential transcript accumulation levels in midgut samples in comparison to the non-infected control [16]. Specifically, two of these genes (AAEL001702 [unknown function] and AAEL001054 [GSTD4]) showed progressively higher transcript accumulation levels in DENV2 samples from 1 to 14 days post-infection (dpi). However, in all these studies, the viruses had been administered to the mosquitoes either along with a bloodmeal or via intrathoracic injections [16–23]. Moreover, most whole-transcriptome analyses in *Ae. aegypti* were performed in context with flavivirus infections, particularly DENV [16, 21–23]. CHIKV, an alphavirus (*Togaviridae*) of the Semliki forest serocomplex, has a particularly short extrinsic incubation period and high infection/dissemination rates in *Ae. aegypti* [5, 27]. Already within 2 days post-oral acquisition, the virus can build up relatively high titers in midgut epithelial cells and, following its dissemination from the midgut, in infected secondary tissues as well.

In this study, we established an alternative, blood-free method of delivering CHIKV to *Ae. aegypti* by feeding a mixture of infectious cell culture, BSA (PM) or PBS (SM) solution, and adenosine triphosphate (ATP). We conducted a genome-wide transcriptome analysis to reveal gene expression patterns in midguts of PM and SM (containing/not containing CHIKV) fed mosquitoes in comparison to a sugarfed control. Eventually, we analyzed in vitro the detailed expression patterns and catalytic activities of two selected candidate genes, *Ae. aegypti* late trypsin (*AeLT*) and serine collagenase 1 precursor (*AeSP1*), in an attempt to reveal their functions.

## Results

### CHIKV orally acquired along with a PM or SM productively infected the midgut and disseminated to secondary tissues

**Fig. 1:**
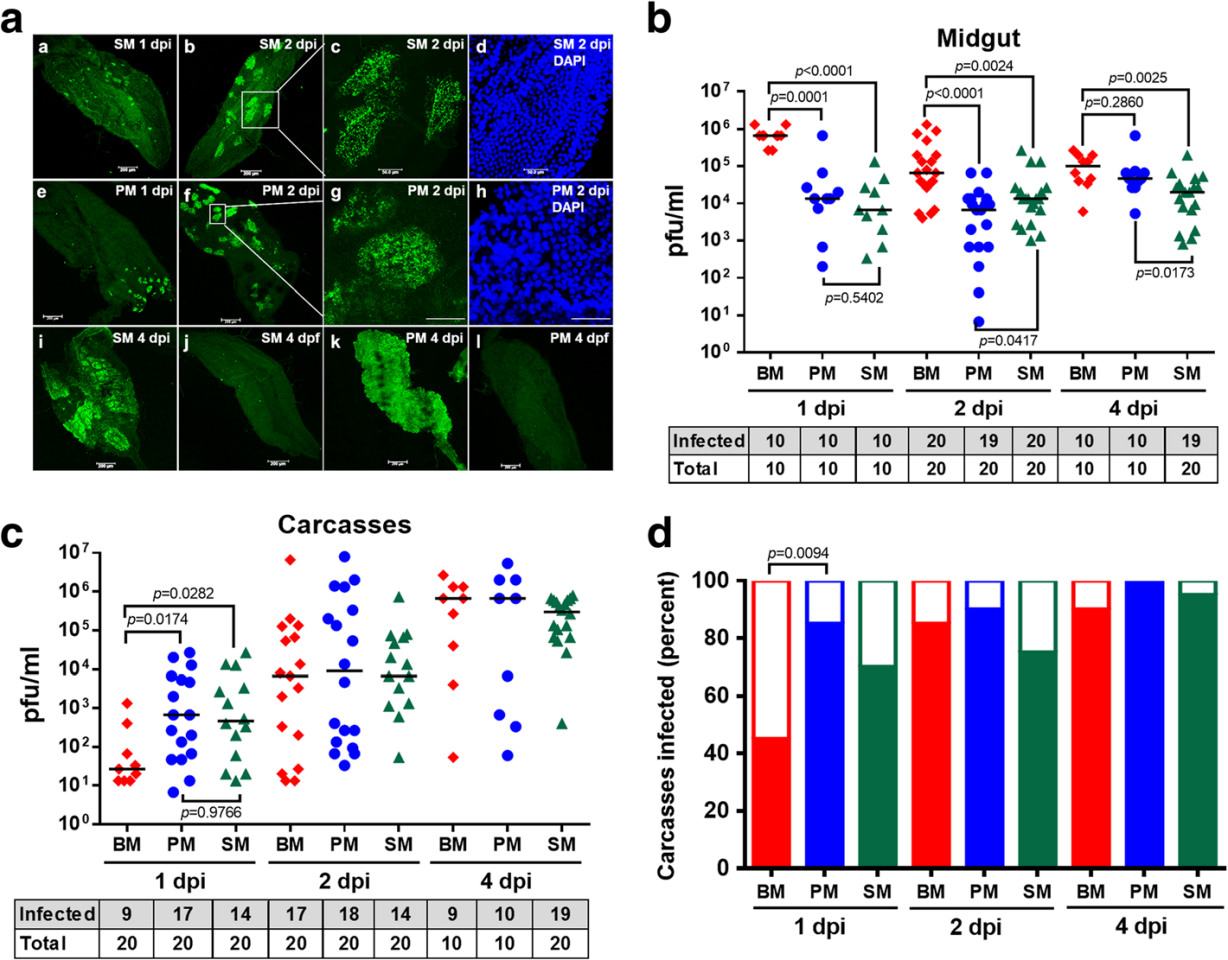
Comparison of CHIKV infection patterns and titers in midguts and carcasses of mosquitoes, which had received a bloodmeal (BM), protein meal (PM), or saline meal (SM) mixed with CHIKV containing cell culture medium at 1, 2, 4 days post-infection (dpi). **a** Immunofluorescence assay detection of CHIKV-antigen in midguts at different time points post-infectious SM/PM (1, 2, 4 dpi) and non-infectious SM/PM (negative control) at 4 days post-feeding (dpf). Images (*c-d*) and (*g-h*) are higher magnification views of (*b*) and (*f*), respectively as outlined by the white boxes. Bars: (*a-b, e-f, i-l*) 200 μm; (*c-d, g-h*) 50 μm. Virus titers of individual midguts (**b**) and carcasses (**c**) as analyzed by plaque assays in Vero cells. Each data point represents the CHIKV titer of a single midgut or carcass. Only infected mosquitoes were included in the statistical analysis based on the Mann–Whitney U-test to determine *P* values. Black bars indicate medians. The tables below **b** and **c** show the numbers of CHIKV infected midguts or carcasses in relation to the total numbers of midgut and carcass samples tested. **d** Prevalence of CHIKV infection in carcasses of mosquitoes. Fisher’s exact test was used to determine *P* values

*Ae. aegypti* (strain HWE) mosquitoes were fed with a (i) blood meal (BM), (ii) 40% BSA solution (PM), or (iii) 2x PBS solution (SM), each diluted 1:1 with cell culture supernatant collected from CHIKV infected (virus titer: 10^7^ pfu/ml) or uninfected Vero cells. All three meal types were deposited in the midgut lumen of the mosquitoes for digestion, whereas the sugarmeal (control) was deposited in the crop and therefore did not enter the midgut. SM was digested at a faster rate (within 24 h after feeding) than PM or BM. Interestingly, SM feeding did not lead to peritrophic matrix formation in the midgut as observed in ultrastructural studies (data not shown). However, all three meal types allowed CHIKV to efficiently infect the midgut epithelium as early as 1 dpi (Fig. 1a-b) and to disseminate from the midgut within 1 dpi as shown by immunofluorescence assays and plaque assays (Fig. 1c-d). CHIKV-containing SM and PM feeding led to the development of distinct viral infection foci in the mosquito midgut at 1 and 2 dpi, which by 4 dpi encompassed most of the midgut tissue (Fig. 1a). Plaque assays showed that overall titers in midguts were significantly lower for CHIKV acquired along with SM or PM in comparison to BM. Surprisingly, CHIKV titers in carcasses (whole mosquito bodies from which midguts had been removed) were significantly higher for PM and SM acquired virus in comparison to BM acquired virus at 1 dpi (Fig. 1c) and significantly higher dissemination rates were observed for PM acquired CHIKV (Fig. 1d). In summary, all three meal types supported the midgut infection with CHIKV and its dissemination to secondary tissues.

### Global changes in the midgut transcriptome in response to sugarfeeding, SM, PM, and CHIKV infection

A total of 27 RNA-Seq libraries from midguts of mosquitoes that had received PM or SM containing/not containing CHIKV at 1 and 2 dpi/days post-feeding (dpf) were generated and sequenced. Libraries from midguts of sugarfed mosquitoes were used as control. Since the BM associated midgut transcriptome of *Ae. aegypti* has been already reported [16, 18, 28], we decided to not include BM in our study. Each experimental variant consisted of three biological replicates (Table 1). RNA sequencing (Illumina) generated on average 34.5 million quality reads per RNA-Seq library of which ~89.2% mapped to the reference genome (Additional file 1). Of 14,728 protein encoding genes of *Ae. aegypti*, 86–89% of them were detected as transcripts in the different midgut RNA-Seq libraries (Table 1). Around 50% of those genes showed a low read coverage (1–10 FPKM) and less than 1% of them had read coverages exceeding 10,000 FPKM.

**Table 1:** Classification of genes based on transcript accumulation levels (at FPKM) in mosquito midguts

Days post- feeding	Meal type	Culture medium	Library	>10,000	1000–10,000	100–1000	50–100	20–50	10–20	1–10	0–1	0
/	Sugar	/	S	12	341	1281	957	1650	1486	3819	3346	1836
1d	SM	Uninfected	P1	55	271	1285	963	1654	1479	3726	3301	1994
	SM	Infected	P1i	60	316	1347	956	1650	1457	3706	3245	1991
	PM	Uninfected	B1	67	376	1387	892	1486	1328	3951	3372	1869
	PM	Infected	B1i	57	313	1217	820	1389	1261	4107	3485	2079
2d	SM	Uninfected	P2	59	261	1238	927	1594	1504	3786	3253	2106
	SM	Infected	P2i	58	283	1276	938	1632	1486	3760	3176	2119
	PM	Uninfected	B2	62	384	1792	1176	1945	1505	3118	3159	1587
	PM	Infected	B2i	63	379	1763	1131	2291	1188	3140	3132	1641

FPKM are average values from RNA-Seq libraries of three biological replicates. SM, saline meal (PBS); PM, protein meal (BSA)

**Fig. 2:**
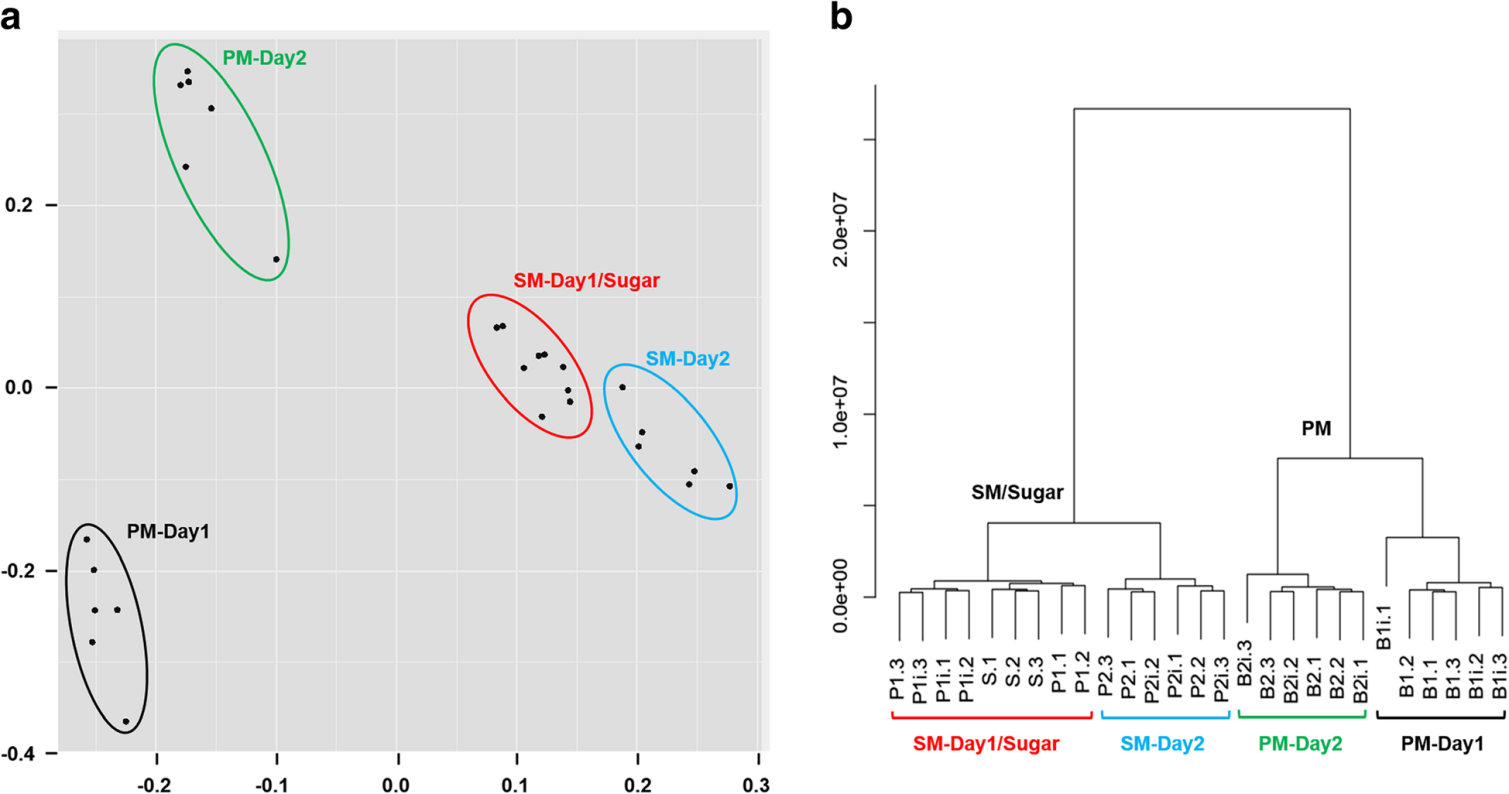
Principal component analysis (PCA) (**a**) and hierarchical cluster analysis (**b**) of gene expression variation among 27 RNA-Seq libraries obtained from midguts of sugarfed, SM fed, and PM fed mosquitoes. For PCA, data visualization and plotting were performed using *R*. The Euclidean distance measure was used to calculate distances and data fitting was performed by Ward's method [55]. SM, saline meal (PBS); PM, protein meal (BSA)

Abundances of reads mapped to genes were quantified by featureCounts and the read counts of all genes across all samples were subjected to principal component analysis (PCA) and hierarchical clustering (Fig. 2). As a result of PCA, the 27 libraries were divided into four large clusters: SM-Day1/Sugar, SM-Day2, PM-Day1, and PM-Day2 (Fig. 2a). Of these four groups, PM-Day1 and PM-Day2 showed the steepest contrast from each other, whereas SM-Day1/Sugar and SM-Day2 were more similar, with data values within the cluster SM-Day1/Sugar only weakly contributing to component variation. Hierarchical clustering revealed two major clusters, SM/Sugar and PM, which were then subdivided according to the PCA results (Fig. 2b). Based on the branch height (shown on the y-axis of the dendrogram) and the PCA clustering, changes in gene expression levels were far less pronounced in midguts of sugarfed/SM fed females than in midguts of PM fed mosquitoes between 1 and 2 dpf/pi. Together, these results suggest that PM ingestion may have caused more dramatic transcriptional changes in the mosquito midgut over time than SM ingestion.

### Expression profiles of DE genes in response to CHIKV infection of the mosquito midgut

**Fig. 3:**
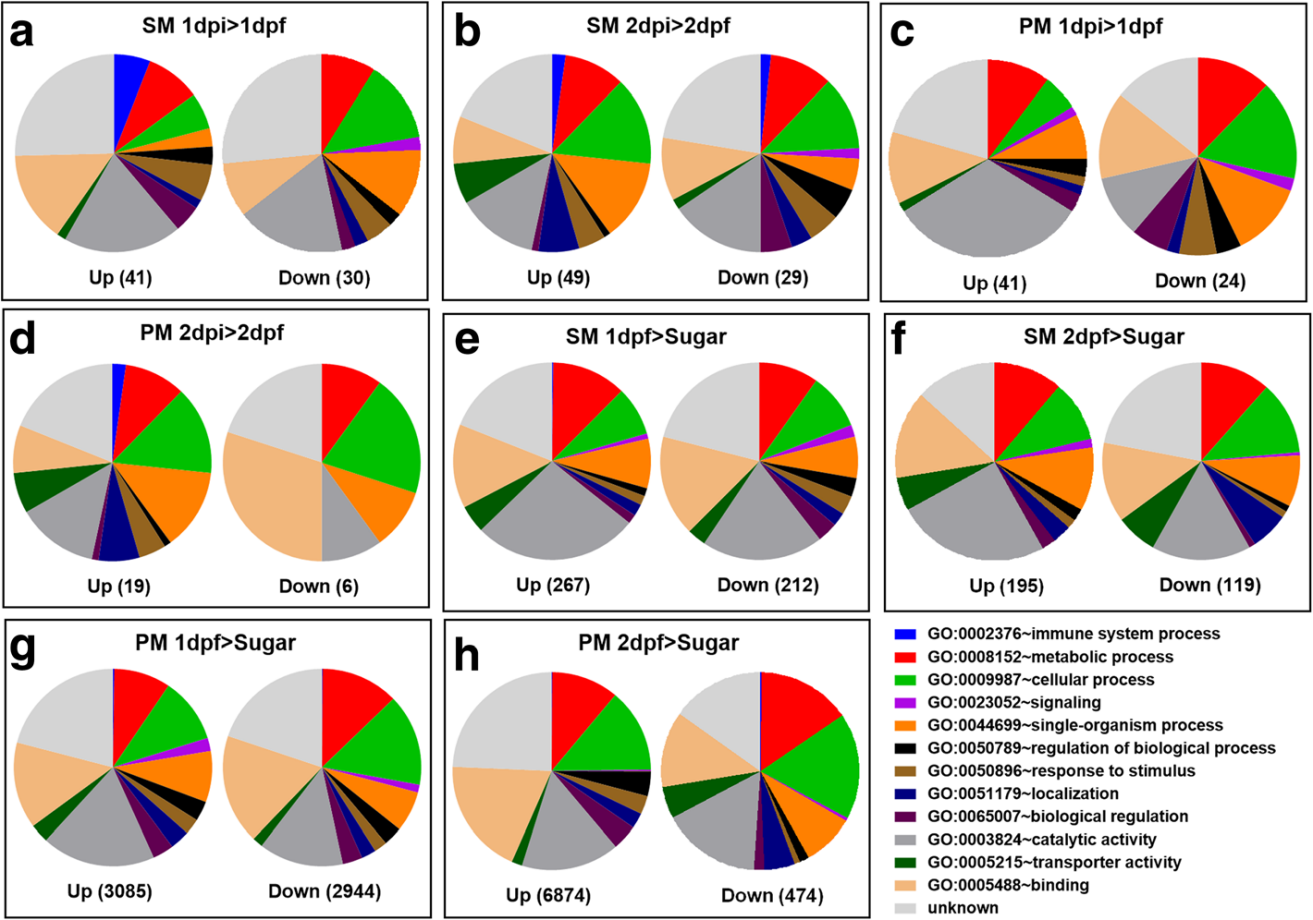
Overview of the functional categories of differentially expressed (DE) genes in response to CHIKV infection (**a**-**d**) and SM/PM feeding (**e**-**h**). DE genes were determined based on statistical analysis by edgeRrobust. The total number of DE genes for each comparison is shown in parentheses in each figure. Gene ontology analysis of DE genes was performed using DAVID Bioinformatics Resources 6.8, and pie charts were generated using GraphPad Prism 6.0. *Up*, upregulated DE genes; *Down*, downregulated DE genes; SM, saline meal (PBS); PM, protein meal (BSA)

Similar to previous reports demonstrating that arboviruses only induced a relatively low number of differentially expressed (DE) genes when acquired along with a BM, we observed only 71 DE genes (41 upregulated and 30 downregulated) at 1 dpi and 78 DE genes (49 upregulated and 29 downregulated) at 2 dpi in midguts of SM fed mosquitoes responding to oral challenge with CHIKV (Additional file 2: Figure S1A, B; Fig. 3a, b; Additional file 3). Based on gene ontology (GO) terms, the majority of these DE genes matched the functional categories *Metabolic Process*, *Catalytic Activity, Response to Stimulus*, *Immune System Process*, *Cellular Process*, *Single-Organism Process*, and *Binding* (Fig. 3; Additional files 4 and 5). An obvious change in the composition of the functional GO categories was caused by the appearance of increasingly abundant transcripts belonging to the category *Immune System Process* in midguts of SM fed, CHIKV infected mosquitoes at 1 and 2 dpi (Fig. 3a, b) and in midguts of PM fed, CHIKV infected mosquitoes at 2 dpi (Fig. 3d). Other profound changes among the RNA-Seq libraries generated from the CHIKV infected midguts were observed for the categories *Transporter Activity*, *Localization*, *Regulation of Biological Processes*, *Biological Regulation*, and *Signaling*. The median logarithmic fold-change (logFC) value for upregulated genes in response to CHIKV infection was 0.832 for 1 dpi and 2.033 for 2 dpi, and that for downregulated genes was 0.971 for 1 dpi and 0.874 for 2 dpi (Additional file 2: Figure S2; Additional file 3). In midguts of PM fed mosquitoes, 65 DE genes (41 upregulated and 24 downregulated) at 1 dpi and 25 DE genes (19 upregulated and 6 downregulated) at 2 dpi were responsive to the presence of CHIKV (Additional file 2: Figure S1A; Fig. 3c, d; Additional file 3). The majority of these DE genes matched the same functional categories as observed for DE genes in CHIKV infected midguts of SM fed mosquitoes (Fig. 3a, b; Additional files 4 and 5). The median logFC value for upregulated genes in the PM RNA-Seq libraries in response to CHIKV infection was 1.234 for 1 dpi and 1.718 for 2 dpi, and that for downregulated genes was 1.604 for 1 dpi and 2.346 for 2 dpi (Additional file 2: Figure S2; Additional file 3). Interestingly, only seven DE genes were identical among the RNA-Seq libraries obtained from CHIKV infected midguts of SM and PM fed mosquitoes at day 1 and 2 (Additional file 3, Additional file 2: Figure S1B). This suggests that the presence of CHIKV induced specific gene expression patterns for each of the different treatments.

### Expression profiles of DE genes related to immunity in response to CHIKV infection and their putative network interactions

According to a heatmap based on FPKM values, >50% and >80% of over 100 immunity related DE genes were upregulated in midguts of PM fed mosquitoes at 1 and 2 dpf/pi, respectively, but not in those of SM fed mosquitoes (Additional file 2: Figure S3A; Additional file 6). Surprisingly, the majority of these immunity related genes were significantly upregulated when CHIKV was absent (Additional file 7), suggesting that ingestion of non-viral protein can induce an immune response in the mosquito midgut. However, in midguts of SM fed mosquitoes at day 1, only nine DE genes related to immunity were significantly upregulated in response to CHIKV (Table 2). These included genes encoding five anti-microbial peptides (AMPs), three fibrinogen-related proteins (FREP), and a peptidoglycan recognition protein (PGRP). At day 2 post-infection with CHIKV, seven immunity related genes were significantly upregulated encoding three AMPs, a thioester-containing protein (TEP), a C-type Lysozyme, a gram-negative binding protein (GNBP), and a FREP (Table 2; Additional file 3). In midguts of PM fed mosquitoes, only one DE gene related to innate immunity, which encoded a GNBP was responsive to the presence of CHIKV at 1 dpi and none at 2 dpi.

**Table 2:** Immunity related genes significantly upregulated in the presence of CHIKV

Libraries	Gene description	Gene ID	logFC	logCPM	*P *Value	FDR
SM 1dpi > 1dpf	Attacin	AAEL003389	7.8160	−0.6624	<0.0001	0.0017
	Cecropin A	AAEL000627	5.4855	−0.9914	0.00015	0.0354
	Cecropin	AAEL015515	4.2884	1.1236	<0.0001	0.0001
	Defensin	AAEL003832	4.9008	−0.5482	<0.0001	0.0004
	Gambicin	AAEL004522	0.6632	7.1876	0.00023	0.0487
	PGRP S1	AAEL009474	1.2361	2.6771	0.00024	0.0495
	FREP 14	AAEL007942	1.2401	4.7265	<0.0001	<0.0001
	FREP 9	AAEL004156	0.6374	6.4320	0.00011	0.0267
	FREP 37	AAEL011007	0.6026	5.8483	<0.0001	0.0242
SM 2dpi > 2dpf	Cecropin	AAEL000621	3.6724	1.1756	<0.0001	0.0007
	Cecropin	AAEL018349	3.2777	0.9248	<0.0001	0.0007
	Defensin D	AAEL003857	2.2313	2.0919	<0.0001	0.0049
	FREP 10	AAEL008646	2.3248	3.8649	<0.0001	0.0000
	Lysozyme C-type	AAEL003723	1.5102	5.0339	<0.0001	<0.0001
	TEP 20	AAEL001794	1.7125	0.2464	0.0002	0.0425
	GNBP	AAEL009178	1.3002	6.4798	<0.0001	<0.0001
PM 1dpi > 1dpf	GNBP	AAEL009178	0.7611	8.7899	0.0001	0.0287

*logFC* logarithmic fold-change, *CPM* counts per million, *FDR* false discovery rate, *PGRP* Peptidoglycan Recognition Protein, *FREP* Fibrinogen Related Protein, *GNBP* Gram-Negative Binding Protein, *SM* saline meal (PBS), *PM* protein meal (BSA), *dpi* days post-infection with CHIKV, *dpf* days post-feeding without CHIKV infection

**Fig. 4:**
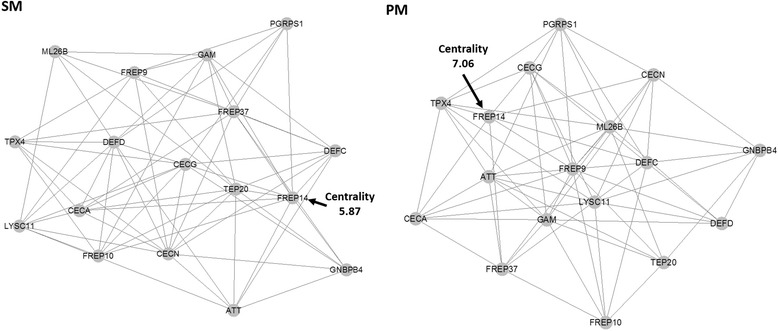
Maximum Relevance Minimum Redundancy (MRMR) expression network of immunity related genes responding to CHIKV infection in midguts of SM and PM fed mosquitoes. The arrows indicate that *FREP14* was predicted as the most central immunity gene in CHIKV infected midguts of SM and PM fed mosquitoes when compared to non-infected midguts demonstrating its importance in the innate immune response to CHIKV infection of the mosquito midgut. The centrality score indicated is based on a degree centrality measure, showing how central the gene is to the given network. Differences in the centrality scores underline their role in differentially modulating the cross-talk of immunity genes depending on SM or PM ingestion. Genes used for the network analysis were: *FREP14* (AAEL007942), *FREP10* (AAEL008646), *GNBP4* (AAEL009178), *PGRP1* (AAEL009474), *FREP37* (AAEL011007), *ML26B* (AAEL013835), *TEP20* (AAEL001794), *CECG* (AAEL015515), *TPX4* (AAEL002309), *ATT* (AAEL003389), *LYSC11* (AAEL003723), *DEFC* (AAEL003832), *DEFD* (AAEL003857), *FREP9* (AAEL004156), *GAM* (AAEL004522), *CECN* (AAEL000621), and *CECA* (AAEL000627)

The Maximum Relevance Minimum Redundancy network analysis showed interactions of these immunity related genes in response to CHIKV infection (Fig. 4). By implementing degree centrality statistics to predict key players within gene expression networks, we predicted a member of the FREP family (FREP14; AAEL007942) as a key player in establishing cross-talks among the immunity gene networks in response to CHIKV infection of SM and PM fed mosquitoes. FREPs are known to play an important role in *Anopheles* as well as in *Aedes* mosquitoes by triggering infection related immune responses [29, 30]. *FREP14* was predicted as the most central of all immunity genes in CHIKV infected midguts of SM and PM fed mosquitoes. The variation of the centrality score for FREP14 between midgut RNA-Seq libraries of (CHIKV infected) SM and PM fed mosquitoes indicates that the diet taken up by the midgut affected the gene's role in differentially modulating the cross-talk of immunity related genes.

### Expression of RNA interference and apoptotic pathway related genes

None of the RNAi or apoptotic pathway related genes showed any significant DE values in any of the libraries irrespective of the presence or absence of CHIKV (Additional files 3 and 6). Based on FPKM values, uniform expression patterns were observed in all RNA-Seq libraries for 15 RNAi pathway-related genes: with the exceptions of *Piwi1* (AAEL008076), *TSN* (AAEL000293), and *Ago2* (AAEL017251), all RNAi related genes were upregulated in CHIKV infected and non-infected midguts of PM fed mosquitoes at 2 dpf/dpi (Additional file 2: Figure S3B). Curiously, 1 day earlier, most of these genes were downregulated, whereas in the SM RNA-Seq libraries their expression levels did not change. When analyzing 22 apoptosis-related genes, the overall expression pattern occurred to be reversed. Only *CASPS18* (AAEL003439) and an inhibitor of apoptosis-like protein encoding gene (AAEL012512) showed upregulation in any of the PM RNA-Seq libraries whereas all other apoptosis-related genes were upregulated in the SM RNA-Seq libraries (Additional file 2: Figure S3C). CASPS18 was described as a putative sensor or enhancer for CASPS19 as the former is lacking its own catalytic activity [31]. However, *CASPS19* was not upregulated in the PM RNA-Seq libraries. Of all caspases, *CASPS16* exhibited the strongest overall gene expression levels (based on FPKM values) followed by *CASPS21*.

Thus, in presence of SM there was a tendency for an apoptotic response whereas PM ingestion had the tendency to trigger RNAi and other immune responses.

### DE genes in midguts of SM and PM fed mosquitoes in comparison to midguts of sugarfed mosquitoes

**Fig. 5:**
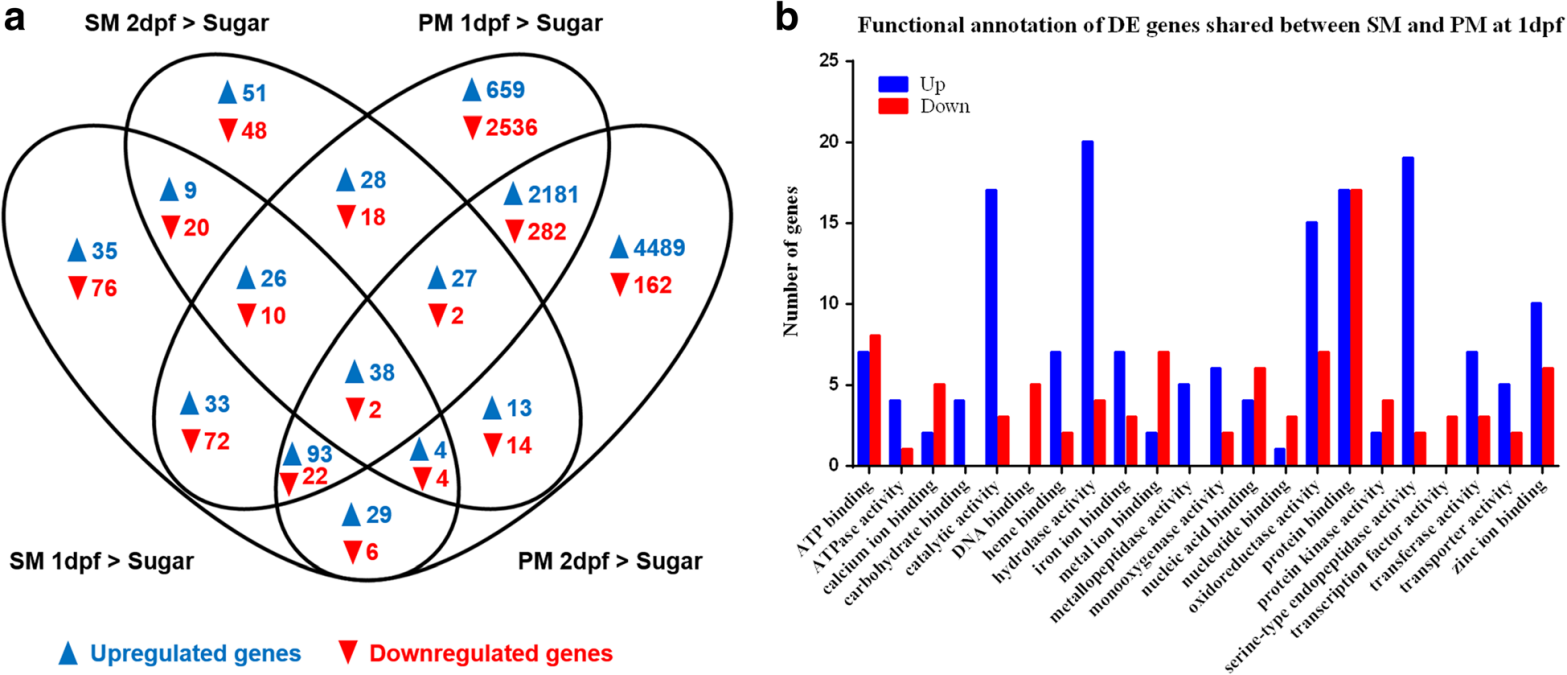
Differential expressed (DE) genes induced by SM and PM at 1 and 2 days post-feeding (dpf) and functional annotation of DE genes at 1 dpf. **a** Venn diagram representing shared DE genes between sugarfed controls and SM/PM samples at 1 and 2 dpf. **b** Histogram presentation of functional categories (as annotated in Vectorbase) of upregulated (*Up*) and downregulated (*Down*) DE genes shared between SM and PM samples at 1 dpf. Gene ontology was analyzed using BioMart at Vectorbase. The x-axis denotes the number of genes in a functional category

SM feeding induced in midguts 479 DE genes (267 upregulated and 212 downregulated) at day 1 and 314 DE genes (195 upregulated and 119 downregulated) at day 2 when compared to midguts of sugarfed mosquitoes (Figs. 3e, f and 5a; Additional file 7). The median logFC value for upregulated genes in response to SM feeding was 0.734 for day 1 and 0.712 for day 2, whereas that for downregulated genes was 0.759 for day 1 and 0.897 for day 2 (Additional file 2: Figure S2; Additional file 7). However, PM ingestion induced a considerably higher number of DE genes, 6029 genes (3085 upregulated and 2944 downregulated) at day 1 and 7368 genes (6874 upregulated and 494 downregulated) at day 2 when compared to midguts of sugarfed mosquitoes (Figs. 3g, h and 5a; Additional files 7, 8 and 9). The median logFC value for upregulated genes in response to PM feeding was 1.261 for day 1 and 0.954 for day 2, whereas that for downregulated genes was 1.010 for day 1 and 0.8420 for day 2 (Additional file 2: Figure S2; Additional file 7).

Similar to the functional categories revealed for DE genes in response to CHIKV infection, DE genes in response to SM or PM feeding belonged to the six largest categories *Unknown* > *Catalytic Activity* > *Cellular Process* > *Binding* > *Metabolic* > *Single Organism Process* (Fig. 3e-h). Profound changes in transcript abundance between SM and PM fed mosquitoes and between 1 and 2 dpf occurred for the GO categories *Transporter Activity*, *Response and Stimulus*, *Regulation of Biological Processes*, *Localization*, *Signaling*, and *Single Organism Process*.

At day 1, SM and PM RNA-Seq libraries shared 190 upregulated DE genes and 106 downregulated genes (Fig. 5a). One day later, the number of commonly upregulated DE genes decreased by about half to 82 genes and that of commonly downregulated genes to just 22. The SM libraries of day 1 and 2 had nine upregulated and 20 downregulated DE genes in common. PM had by far the strongest effect on DE gene upregulation in the midgut. Of those upregulated DE genes, 2181 were commonly expressed at 1 and 2 dpf and both libraries shared 282 downregulated DE genes. The profound increase in the number of upregulated genes (from dozens to thousands of genes) in the PM libraries when compared to SM libraries suggests that most of these genes were involved in protein (BSA) digestion. Common DE genes among the SM and PM RNA-Seq libraries obtained from midguts of non-infected mosquitoes at 1 dpf belonged to the categories *Catalytic Activity*, *Hydrolase Activity*, *Serine-Type Endopeptidase Activity*, *Metalloproteinase Activity*, *Protein Binding*, and *Oxidoreductase Activity*, three of which (*Catalytic Activity*, *Serine-Type Endopeptidase Activity,* and *Metalloproteinase Activity*) may contain proteinase genes involved in BL degradation and remodeling (Fig. 5b).

### Comparing expression levels of genes encoding trypsins, metalloproteinases, and serine-type endopeptidases

**Fig. 6:**
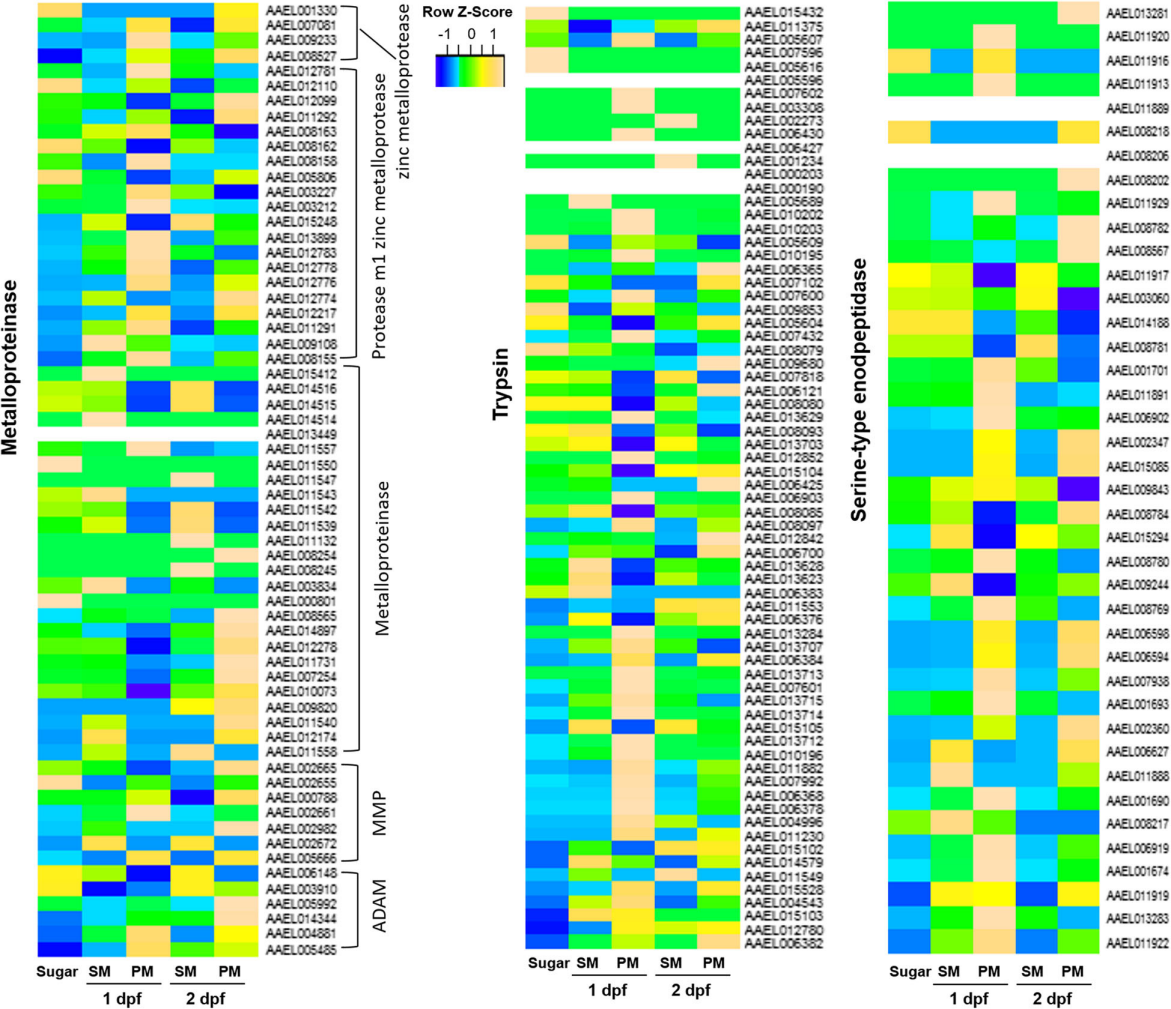
Median-normalized expression levels (at FPKM) of three groups of proteinase genes (metalloproteinases, trypsins, and serine-type endopeptidases). Data show a comparison between sugarfed controls and SM/PM samples at 1 and 2 days post-feeding (dpf) and are presented as heatmaps for each transcript (*vertical axis*) at each time point (*horizontal axis*), with *yellow* and *blue* indicating high and low levels of expression, respectively

In view of their possible involvement in BL degradation and remodeling, we focused on three groups of proteinase genes: trypsins, metalloproteinases, and serine-type endopeptidases whose relative expression levels are presented in heatmaps based on FPKM values (Fig. 6; Additional file 10). SM or PM ingestion caused increased expression of >50% of the trypsin, metalloproteinase and serine-type endopeptidase encoding genes, including genes for two MMPs, AeMMP1 (AAEL005666) and AeMMP5 (AAEL002661), two ADAMs/ADAMTS (AAEL005485, AAEL014344), and two predicted serine collagenases (AAEL013284, AAEL007432). As a general trend, most of the trypsin, metalloproteinase, and serine-type endopeptidase genes showed upregulation in the PM RNA-Seq libraries in comparison to the sugarfed library. Ten, seven, and four DE genes encoding trypsins, serine-type endopeptidases, and metalloproteinases, respectively, were significantly upregulated in midguts of SM or PM fed mosquitoes at 1 dpf (Table 3). In addition, two serine collagenase-like genes and one glutamate carboxypeptidase encoding gene were identified. Most of these genes were highly expressed in midguts and their expression levels were increased by logFC > 2 in SM and logFC > 5 in PM.

**Table 3:** Proteinase genes significantly upregulated in the presence of SM and PM at 1 dpf in comparison to sugarfeeding

Gene description	Gene ID	SM > Sugar 1 dpf			PM > Sugar 1 dpf		
		logFC	logCPM	FDR	logFC	logCPM	FDR
Serine-type endopeptidase	AAEL001674	2.4369	3.7976	<0.0001	9.8809	11.0771	<0.0001
	AAEL001690	2.5570	7.6695	<0.0001	7.6210	12.5213	<0.0001
	AAEL001693	1.0448	6.5114	<0.0001	3.3007	8.1254	<0.0001
	AAEL002360	1.1791	5.5518	<0.0001	6.0935	9.9537	<0.0001
	AAEL006594	0.9889	4.2450	0.0372	5.9591	8.6937	<0.0001
	AAEL006598	1.5849	2.5658	0.0002	6.2810	6.7162	<0.0001
	AAEL018285	0.7099	2.7168	0.0131	3.4892	4.8222	<0.0001
Trypsin	AAEL007601	1.7085	4.8914	0.0048	5.4613	8.0474	<0.0001
	AAEL010195	2.6617	2.6170	<0.0001	7.6061	7.5845	<0.0001
	AAEL010196	3.1053	6.6037	<0.0001	6.9277	10.3539	<0.0001
	AAEL010202	2.3166	0.1170	0.0372	11.3584	8.5362	<0.0001
	AAEL010203	2.7968	2.8780	<0.0001	8.6558	8.5975	<0.0001
	AAEL013707	1.2571	7.7352	0.0005	1.3679	7.5083	0.0197
	AAEL013713	1.4760	2.6240	0.0005	10.4679	11.0187	<0.0001
	AAEL013715	2.6119	7.1701	<0.0001	2.9509	7.1257	<0.0001
	AAEL013712	2.8643	4.1907	<0.0001	8.9742	10.1751	<0.0001
	AAEL013714	2.8748	6.9939	<0.0001	6.6158	10.3552	<0.0001
Late trypsin	AAEL013284	1.4787	6.0790	<0.0001	10.7835	14.9395	<0.0001
Serine collagenase 1	AAEL007432	2.1355	8.3610	<0.0001	7.4848	13.4289	<0.0001
Glutamate carboxypeptidase	AAEL001588	0.4942	7.0966	0.0009	3.2833	9.1609	<0.0001
M1 zinc metalloprotease	AAEL008163	0.4717	6.5214	0.0017	0.9800	6.7167	<0.0001
	AAEL008155	0.5685	8.4535	0.0004	1.7879	9.2047	<0.0001
	AAEL012783	0.7819	9.8040	0.0022	2.2170	10.7078	<0.0001
	AAEL009108	4.7926	2.4011	<0.0001	2.7697	0.3896	<0.0001

*logFC* logarithmic fold-change, *CPM* counts per million, *FDR* false discovery rate, *SM* saline meal (PBS), *PM* protein meal (BSA), *dpf* days post-feeding without CHIKV infection

MMPs, ADAMs/ADAMTS, and collagenases are known to be involved in extracellular matrix remodeling and tissue repairing [9–12]. It is possible that trypsins, other metalloproteinases, and serine-type endopeptidases are also involved in midgut BL degradation and/or remodeling either directly by taking part in proteinase activation cascades or indirectly by participating in relevant signaling processes [32, 33].

### Assessing the catalytic activities of AeSP1 and AeLT in vitro

**Fig. 7:**
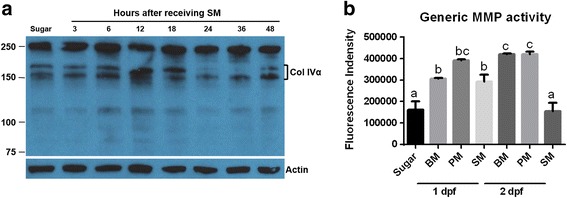
Effects of SM feeding on midgut collagen IV abundance and generic MMP activity. **a** Western blot showing abundance of midgut collagen IV at different time points post-SM feeding using polyclonal antibodies generated against human collagen IV. β-actin was detected as a loading control. Molecular masses in kDa are indicated. **b** Generic MMP activity of midgut lysates prepared from mosquitoes that had received a BM, PM, or SM at 1 and 2 dpf (sugar, control). Mean values with standard deviations (SD) from three independent experiments are shown. Different letters indicate significant differences based on one-way analysis of variance (ANOVA) followed by Tukey's multiple comparisons test (* at *p* ≤ 0.01). BM, blood meal (from sheep); SM, saline meal (PBS); PM, protein meal (BSA)

Being a major component of the midgut BL, we wanted to investigate whether collagen IV abundance was changed in mosquito midguts following SM ingestion. When we conducted a time series study of collagen IV degradation in midguts of SM fed mosquitoes, it became evident that midgut collagen IV was less abundant at 24 and 36 h post-feeding in comparison to other time points (Fig. 7a). Concurrent with midgut collagen IV degradation, generic MMP activity was significantly increased in mosquito midguts at 1 day post-SM (also: post-PM or BM) in comparison to the sugarfed control. No significant difference was observed between midguts of SM and sugarfed mosquitoes at 2 dpf while BM and PM acquisition resulted in a significantly higher MMP activity (Fig. 7b). These results support the conclusion that BM, SM, and PM feeding can stimulate or support BL remodeling by affecting the abundance of collagen IV via an increased MMP activity. The results also suggest that genes encoding proteinases involved in collagen IV degradation/remodeling were expressed in midguts of SM fed mosquitoes. The potential involvement of AeMMPs in midgut BL remodeling has been investigated in a recent study [6].

**Fig. 8:**
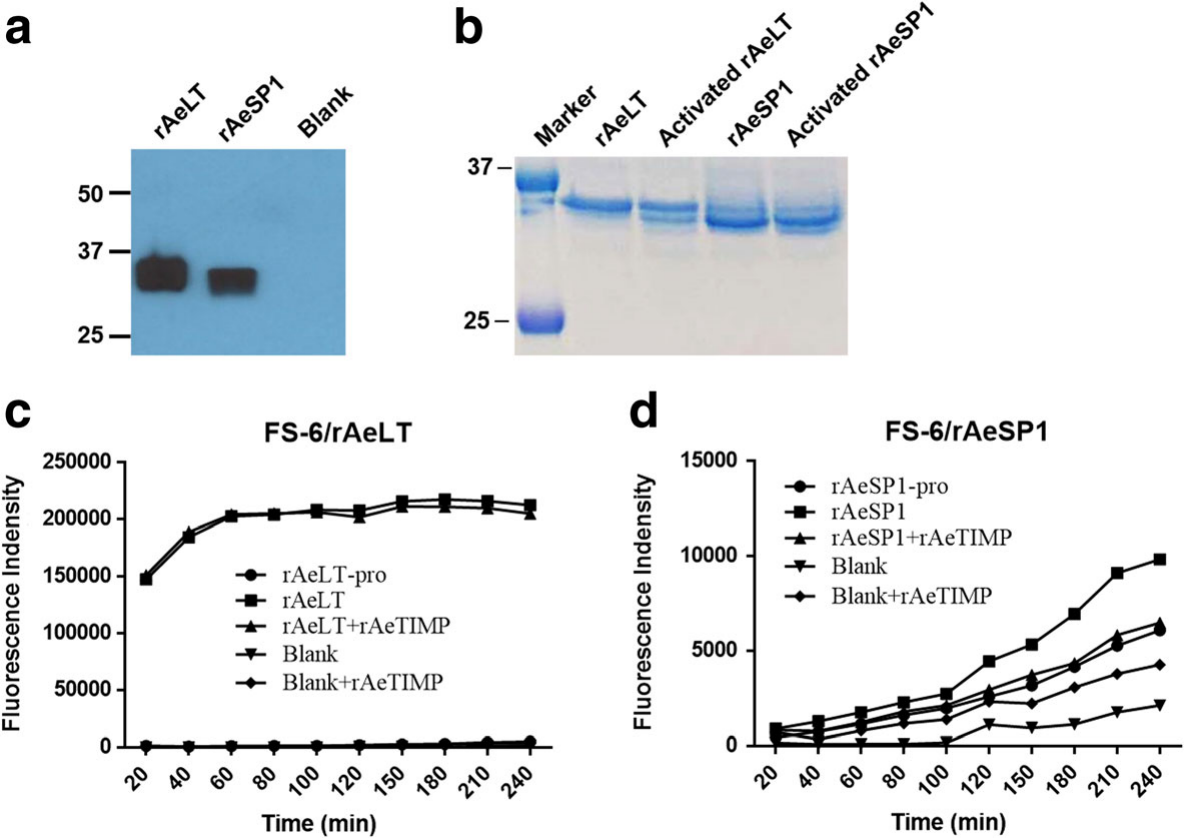
*Drosophila* S2 cell expression of two putative serine collagenase genes and catalytic activities of the recombinant proteins. **a** Western blot detection of recombinant (r)AeLT and (r)AeSP1 proteins using an His tag-specific monoclonal antibody. Blank, S2 cells were transfected with non-insert containing plasmid vector; rAeLT and rAeSP1, S2 cells were transfected with plasmid vectors expressing AeLT or AeSP1. Molecular masses in kDa are indicated. **b** SDS-PAGE showing purified rAeLT and rAeSP1 with or without additional trypsin activation (pro-protein cleavage). Molecular masses in kDa are indicated. Kinetics of rAeLT (**c**) and rAeSP1 (**d**) in vitro activities and their inhibition by rAeTIMP. Twenty ng of purified rAeLT or rAeSP1 were preincubated with/without TPCK-treated trypsin for 15 min at RT, then incubated with 20 ng of rAeTIMP, or reaction buffer (RB) at RT for 2 h, followed by addition of FS-6 substrate. Fluorescence intensity was measured every 20 min. rAeLT-pro and rAeSP1-pro, pro-protein of rAeLT and rAeSP1; rAeLT and rAeSP1, activated rAeLT and rAeSP1

Here, we focus on the potential involvement of two putative collagenase genes, *AeLT* and *AeSP1* in midgut BL degradation/remodeling. As revealed by qRT-PCR, both genes were significantly upregulated in midguts at 1 dpf/pi in conjunction with BM or PM ingestion; however, only *AeLT* was significantly upregulated in SM fed midguts at 1 dpf/pi (Additional file 2: Figure S4). As shown by Western blot analysis, expression of *AeLT* and *AeSP1* in *Drosophila* S2 cells resulted in recombinant proteins with sizes of 35 kDa and ~33 kDa for *AeLT* and *AeSP1*, respectively, when using an anti-His-tag specific monoclonal antibody for detection (Fig. 8a). Following their activation via incubation with TPCK-treated bovine trypsin, both proteins produced band signals that were reduced in size by ~2 kDa indicating that pro-domain cleavage had occurred (Fig. 8b). In vitro, activated recombinant (r)AeLT showed strong generic MMP activity using FS-6 as substrate (Fig. 8c) whereas rAeSP1 did not (Fig. 8d). Further, addition of the recombinant *Aedes aegypti* tissue inhibitor of metalloproteinases (rAeTIMP) did not inhibit rAeLT activity indicating that the catalytic domain of the proteinase is structurally different from that of MMPs or ADAMs both of which can be inhibited by TIMPs [34, 35].

.

## Discussion

Bloodmeal uptake by the mosquito midgut leads to a global upregulation of endogenous genes, many of which encode proteins specifically involved in bloodmeal digestion [18, 28]. Thus, offering mosquitoes minimal meals lacking any substantial nutritional value seemed to be a promising strategy to limit gene expression to those candidate genes responding to structural changes in the midgut due to tissue expansion rather than to genes involved in digestion. Ingestion of PM, which consisted of BSA and this way was of nutritional value, caused upregulation of several thousand genes making this meal type physiologically look similar to a bloodmeal. However, in contrast to blood, BSA is a highly purified protein and free of proteinases, enzymes and growth factors, which all are highly abundant in blood sources. Thus, the midgut transcriptome of PM fed mosquitoes should predominantly reflect any effects associated with feeding, protein digestion, and changes in tissue structure. Replacing protein with a substance without nutritional value such as salt (PBS) in SM, should strongly reduce the expression of genes involved in digestion. In agreement with this hypothesis, we only recorded 267 DE genes that were upregulated by SM at 1 dpf, compared to 3085 genes which were upregulated in presence of PM at 1 dpf or 4160 genes upregulated at 5 h post-BM (in whole-body mosquitoes) [28]. The presence of CHIKV in SM induced expression of 149 DE genes in total at 1 and 2 dpi, which was similar to the number of genes observed to be upregulated in the CHIKV containing PM libraries (140 DE genes). To compare with another study, DENV2 induced expression of 31, 96, and 14 DE genes in midguts of *Ae. aegypti* at 1, 4 and 14 dpi via BM, respectively [16]. Most of the DE genes responding to CHIKV challenge along with SM feeding had been previously reported to be responsive to arboviruses such as DENV2, WNV and YFV [16, 22, 23, 26]. Interestingly, ingestion of CHIKV along with SM or PM significantly reduced the midgut infection intensity of the virus but increased its carcass infection intensity in comparison to BM acquired CHIKV. These observations support the conclusion that blood may contain additional components that are advantageous for viral cell entry.

Our data show that CHIKV induced expression of numerous genes related to immunity including those encoding AMP, FREP and PDGR. We identified additional genes (not related to immunity) that were significantly upregulated in the presence of CHIKV and had previously been reported to be involved in arbovirus infection of mosquitoes. These genes encoded three synaptic vesicle (SV) proteins (AAEL005849, AAEL002294, and AAEL007489), UNC93A (AAEL004048), cysteine-rich venom protein (CRVP; AAEL008473), and two C-type lectins (CTL) (AAEL011404 and AAEL011407). In mammals, UNC93A and SV proteins are involved in intracellular transport [36] and their transcripts were enriched more than 40-fold during Sindbis virus (SINV; *Alphavirus*; *Togaviridae*) or DENV2 infection of *Ae. aegypti* [37, 38]. UNC93A and SV seem to play a role in virus assembly and/or budding from the infected cell as revealed by transient gene silencing of *SV2* or *UNC93A* in mosquitoes infected with SINV [37]. CRVP proteins are known to contain a trypsin inhibitor-like (TIL) domain, and therefore could be functioning as serine protease inhibitors [39]. One of the *Ae. aegypti* CRVP genes*, CRVP379* (AAEL000379), has recently been reported to be significantly upregulated in mosquitoes infected with the flaviviruses YFV or WNV. Further, *CRVP379* expression proved to be essential during infection of *Ae. aegypti* mosquitoes or Aag2 cells with DENV2 [40]. CTLs are a family of proteins possessing carbohydrate-binding activity. Several mosquito CTLs have been reported to be interacting with arboviruses as ligands to facilitate mosquito infection [41, 42]. CTLs play a role in maintaining gut microbiome homeostasis by manipulating the bactericidal capacity of AMPs [43]. In *Ae. aegypti*, the galactose specific C-type lectin-1 (mosGCTL-1) increased the attachment efficiency of WNV to the cell membrane via binding to a membrane-bound protein (mosPTP-1) [42]. Furthermore, mosGCTL-3 enhanced DENV2 infection via interacting with the E protein of the virus [41]. Besides those genes whose possible functions during arbovirus infections have been analyzed by others, we also observed numerous genes to be significantly upregulated in presence of CHIKV, whose functions do not appear to be directly related to virus infection. These genes, for example, include a cytochrome P450 gene (AAEL006811), two lethal(2)essential for life (l(2)efl) genes (AAEL013352, AAEL013349), a juvenile hormone-inducible protein gene (AAEL006607), a heat shock protein gene (AAEL013350), and a microtubule binding protein gene (AAEL013242).

In a recent study in which we compared the vector competence of two *Ae. aegypti* strains (ORL and HWE) for CHIKV, we suggested that the virus exits the midgut tissue by crossing the BL surrounding the organ instead of using tracheal cells or the cardia as a conduit for dissemination [5, 44]. In agreement with this finding, we observed here that the abundance of collagen IV, a major component of the BL, was diminished between 18–48 h post-SM, which coincided with an increased generic MMP activity. In eukaryotes, BL degradation/remodeling involves the activities of numerous metalloproteinases and inhibitors [11, 12] and an aim of this study was to identify potential candidate genes involved in mosquito BL degradation/remodeling. In insects, several MMPs such as *Drosophila* MMP1 and MMP2 have been shown to cleave collagen in vitro [45, 46]. Here, we also observed transcriptional upregulation of two MMPs, *AeMMP1*and *AeMMP5* [6] and two ADAMs/ADAMTS in response to SM and/or PM ingestion. In addition, two putative serine collagenase genes were significantly upregulated in the midgut RNA-Seq libraries of SM and PM fed mosquitoes and their expression patterns were further confirmed by qRT-PCR.

In insects, a serine collagenase was for the first time identified in the heel fly *Hypoderma lineatum* [47] with the purified enzyme being able to degrade native collagen but unable to degrade synthetic peptide substrates specific for trypsin or chymotrypsin [48]. Despite its designation as a trypsin, bacterially expressed recombinant (r)AeLT did not affect cleavage of the trypsin specific substrate DL-BAPNA, whereas transient silencing of *AeLT* had no effect on the midgut infection efficiency of DENV2 suggesting that AeLT may actually lack any trypsin activity [49–51]. Based on structural similarities, AeLT does not resemble a canonical trypsin but a serine collagenase instead [49]. We expressed the full-length *AeLT* in *Drosophila* S2 cells and the resulting rAeLT showed a high level of generic MMP activity. Therefore, our results strongly support the conclusion that AeLT, indeed, is a serine collagenase. In contrast, recombinant (r)AeSP1 lacked any MMP activity, which is not surprising because in AeSP1, His57 and Ser195 in the archetypal His57-Asp102-Ser195 catalytic triad motif are changed to Gln57 and Val195 [49].

## Conclusions

Substituting BM for PM or SM did not affect the persistent infection of *Ae. aegypti* with CHIKV, although virus titers in midguts were significantly lower when the virus was acquired along with SM instead of a bloodmeal. On the other hand, PM and SM did not negatively affect the dissemination rates of the virus, indicating that the protein content of the ingested meal was not a major determinant of CHIKV midgut escape from the midgut. Instead, (over)stretching of the midgut tissue due to meal ingestion may be the critical mechanism or trigger underlying viral midgut escape. Gene expression patterns in PM fed midguts largely resembled those previously observed for bloodfed midguts. The presence of CHIKV in any of the ingested meals had relatively minor effects on the overall gene expression profiles in midguts, as only 232 genes (~1.8% of all detectable transcripts) were being differentially expressed, 17 (~7%) of which were related to immunity. In comparison to PM feeding resulting in the upregulation of 9959 genes at day 1 and 2, SM feeding substantially reduced the gene expression profile in the mosquito midgut with only 462 (~3.5%) genes being significantly upregulated, many of which belonged to the functional category *Catalytic Activity.* These genes included seven serine-type endopeptidases, ten trypsins, two serine collagenases, four M1 zinc metalloproteases, and a glutamate carboxypeptidase, which could be involved in the midgut escape of CHIKV when traversing the midgut BL. The recombinant serine collagenase, AeLT, showed strong generic MMP activity in vitro.

## Methods

### Mosquito samples

*Ae. aegypti* mosquitoes of the Higgs White Eye (HWE) strain, exhibiting eye-pigment deficiency, were reared and maintained in a BSL2 insectary at 28°C, 75–80% relative humidity, and a 12 h light/12 h dark cycle. For colony maintenance, mosquitoes received artificial bloodmeals consisting of defibrinated sheep blood (Colorado Serum Company, Denver, CO).

### CHIKV infection of mosquitoes and virus detection

CHIKV strain 37997 (West African genotype; GenBank accession: AY726732.1) was propagated in Vero cells in T25 flasks at a multiplicity of infection (m.o.i.) of 0.01 using Minimum Essential Medium Eagle (MEM) complemented with 7% FBS. For PBS feeding (SM), Vero cells were prewashed with serum-free MEM medium, and infected with CHIKV in T25 flasks at an m.o.i. of 0.01 using serum-free MEM medium. Virus-infected cells were observed under a microscope until around 70% cells (usually 26–32 h pi) started to float. Thereafter, cell culture media was collected and mixed with an equal amount of defibrinated sheep blood, 40% BSA (Sigma Aldrich, St. Louis, MO) or 2x PBS (pH 7.4, Sigma) including 10 mM ATP. One week-old females that had been deprived of sugar for 24 h were fed for 1 h with a mixture consisting of CHIKV-infected cell culture and blood, BSA, or PBS (or non-infected cell culture mixture as a control) at 37°C using a single glass feeder per carton. Fully engorged females were selected and maintained in 1.9 L (64 oz.) cartons by providing raisins and water until further analysis.

CHIKV antigen was detected by immunofluorescence assays and virus titers of individual midguts and carcasses were determined by plaque assays as described before [5]. All mosquito infections and CHIKV detection assays were carried out in a Biosafety Level 3 laboratory within the Laboratory for Infectious Disease Research (LIDR) of the University of Missouri.

### RNA-Seq library preparation and sequencing

Total RNA was extracted from 15 midguts per sample using TRIzol reagent (Invitrogen, Carlsbad, CA). RNA samples were DNase-treated to remove potential contamination from genomic DNA. RNA purity and concentration were analyzed with a NanoDrop 1000 v 1.3.2 (Thermo Scientific, Wilmington, DE). The absence of RNA degradation was initially assessed by electrophoresis of 1 μg of RNA on a 1.0% agarose gel. Thereafter, the quality of each RNA sample was evaluated using an Agilent 2100 Bioanalyzer (Agilent Technologies, Santa Clara, CA) equipped with an RNA Nano Chip.

mRNA samples were further prepared for RNA-Seq analysis using the TruSeq RNA Sample Preparation Kit (Illumina, San Diego, CA). First-strand cDNA synthesis from the fragmented RNA was carried out via reverse transcription. Following second-strand cDNA synthesis, the resulting double-stranded DNA underwent end-repair and adenylation of its 3’ends. To produce the final sequencing library, universal adapters were ligated to the cDNA fragments followed by PCR amplification. The resulting template molecules were used for sequencing on the Illumina NextSeq 500 instrument with multiple lanes and paired-end read (2 x 75 bases). Each treatment was analyzed as three independent biological replicates.

### RNA-Seq data processing and analysis of differential gene expression

The raw sequences (fastq) were subjected to quality check by FastQC (http://www.bioinformatics.babraham.ac.uk/projects/fastqc/). Adapter removal and quality trimming of the raw reads were performed using fqtrim (https://ccb.jhu.edu/software/fqtrim/). Reads shorter than 30 nucleotides and quality scores less than 25 were excluded from further analysis. The trimmed reads were subjected to mapping to the *Ae. aegypti* reference genome AaegL3 using Hisat2 mapper [52]. We used FeatureCounts [53] to quantify transcript abundance in each sample using the gene annotation AaegL3.3 obtained from VectorBase (https://www.vectorbase.org/organisms/aedes-aegypti). Differential expression (DE) analysis between sample groups was performed by edgeRrobust [54].

### Gene ontology and pathway annotation

Gene ontology and KEGG pathway analyses of DE genes were performed using DAVID Bioinformatics Resources 6.8 (https://david.ncifcrf.gov). Pathway painting was performed using KEGG mapper (http://www.genome.jp/kegg/mapper.html). Venn diagrams were generated using Venny 2.1 (http://bioinfogp.cnb.csic.es/tools/venny/index.html) and heatmaps were generated using scripts for *R*.

### Cluster and network analysis

The expression data of all genes were subjected to hierarchical clustering to generate sample dendrograms. The Euclidean distance measure was used to calculate distance and data fitting was achieved by Ward's method [55]. All principal component analyses (PCA), data visualization and plotting were performed using *R*. The network analysis was performed using ‘minet’ based on mutual information (MI) [56]. MI is a measure of mutual dependences between two variables that infers how much one variable tells us about the other variable. MIs were calculated using the Spearman estimator, with no discretization method applied to the data prior to calculation. The square matrix of all MI values among all genes was then used to generate a weighted adjacency matrix by the Maximum Relevance Minimum Redundancy (MRMR) method [56]. The adjacency matrix was then used to plot the expression networks. Key genes in the networks were predicted from degree centrality scores (predicting how central each gene subset is relative to the network structure) using a ‘key player analysis’ approach, a method used in analyzing social networks [57].

### Recombinant protein expression and purification

Recombinant AeLT and AeSP1 proteins were expressed in *Drosophila* S2 cells. *AeLT* and *AeSP1* coding sequences were amplified via RT-PCR using a forward primer containing a *Kpn*I restriction site and a reverse primer containing an *Xba*I restriction site (Additional file 11). Resulting amplicons were *Kpn*I/*Xba*I digested and inserted into the similarly digested pMT/Bip/V5-His B expression vector (Invitrogen). The resulting recombinant plasmids were transiently transfected into S2 cells using TransFectin Lipid Reagent (Bio-Rad, Hercules, CA). Control cells were transfected with the non-modified pMT/Bip/V5-His B plasmid vector. Recombinant protein production was induced by adding 500 μM CuSO_4_ to serum-free Schneider medium (Lonza, Basel, Switzerland). Samples containing recombinant protein were collected at 2 days after induction and validated by Western blot using an anti-His tag monoclonal antibody (Thermo Scientific). To purify recombinant proteins, cell-free medium (30 ml) was incubated for 3 h under rotation at 4°C with 2 ml of Ni-NTA His-bind resin (Thermo Scientific) equilibrated with binding buffer (20 mM sodium phosphate, pH 7.4, 0.3 M sodium chloride, 10 mM imidazole). Ni-NTA His-bind resin and medium were then packed into an empty spin column (9 cm high and 2 ml bed volume, Bio-Rad) supplied with a 15 ml tube on ice. The column was washed with 60 ml of binding buffer. Bound proteins were eluted in four separate eluates, each having a volume of 0.5 ml of elution buffer (20 mM sodium phosphate, pH 7.4, 0.3 M sodium chloride, 250 mM imidazole). The elutes were analyzed by SDS-PAGE and Western blot using an anti-His tag monoclonal antibody (Thermo Scientific) and fractions containing recombinant protein were then equilibrated and concentrated using Centrifugal Filter Units (10 kDa, Millipore, Billerica, MA).

### Western blots

Midguts were homogenized in 2x Laemmli sample buffer (Bio-Rad), boiled for 5 min, and centrifuged at 10,000 *g* for 10 min. The supernatants were separated by SDS-PAGE and transferred to a nitrocellulose membrane. After blocking with 5% non-fat dried milk in a Tris-buffered saline buffer (20 mM Tris–HCl, 150 mM NaCl, 1 mM EDTA, 0.1% Tween 20, pH 7.5) (TBST) for 1 h, the membrane was incubated in the blocking solution overnight at 4°C with anti-His tag monoclonal antibody (Thermo Scientific) or anti-human collagen IV polyclonal antibodies (Abcam, Cambridge, MA, USA). Following overnight incubation with the primary antibodies, membranes were washed three times (6 min/wash) in TBST. Membranes were incubated with anti-rabbit IgG-HRP or anti-mouse IgG-HRP (Cell Signaling Technology, Danvers, MA, USA) at RT for 2 h and then treated for 1 min with SuperSignal West Pico chemiluminescent substrate (Pierce, Waltham, MA, USA). The immunoreactive proteins were visualized by exposing the membrane to an x-ray film. Anti-β-actin-peroxidase antibody (Sigma) was used to detect the loading control in each lane.

### qRT-PCR

Groups of six midguts were collected for total RNA extraction using Trizol reagent (Invitrogen). First-strand cDNA was synthesized from 1 μg total RNA using QuantiTect Reverse Transcription Kit (Qiagen, Hilden, Germany). Gene specific primers (Additional file 11) were used for qPCR amplification of cDNA samples and the ribosomal S7 protein encoding gene (*RpS7*, AAEL009496) as an endogenous reference. qPCR amplifications and analyses were carried out using an Applied Biosystems (ABI, Warrington, UK) 7300 Real-Time PCR System. The final reaction volume was 20 μl using SYBR green Supermix (ABI). The PCR program was: hold at 95°C for 10 min, then 95°C for 15 s and 60°C for 1 min, repeated for 40 cycles. The specificity of the SYBR green PCR signals was further confirmed by melting curve analyses. Relative abundance of each gene was normalized and calculated against that of *RpS7* as an endogenous reference using the 2^−ΔΔ^C_T_ method. Each sample/treatment was analyzed as three independent biological replicates.

### In vitro activity assays

Groups of six midguts were homogenized in assay buffer (50 mM Tris–HCl, 150 mM NaCl, 5 mM CaCl_2_, pH7.6), and homogenates were centrifuged at 10,000 *g* (4°C) for 10 min. The supernatants were collected and stored at −80°C for activity assays. MMP activity of midgut samples was measured using the generic MMP assay kit (Anaspec, Fremont, CA, USA). Each sample/treatment was analyzed as three independent biological replicates.

Recombinant AeLT and AeSP1 were activated with trypsin according to the method of Tsu and Craik [9]. The activation reaction contained 5 μl of recombinant protein (20 ng), 2.5 μl of 1 mM TPCK-treated bovine trypsin (Sigma) and 2.5 μl of 4x assay buffer. Following activation at RT for 15 min, FS-6 substrate (Millipore) was added to a final concentration of 10 μM in reaction buffer and fluorescence intensity was measured at Ex = 328 nm and Em = 393 nm every 20 min (over 4 h) with a Perkin-Elmer LS50B spectrometer. For rAeTIMP inhibition assay, 20 ng of the active recombinant AeLT and AeSP1 were preincubated with 20 ng of rAeTIMP at RT for 2 h before adding the fluorescent substrate.

## Additional files

Additional file 1: Table S1. Read mapping statistics of samples. (XLSX 10 kb)

Additional file 2: Figures S1–S4. **Figure S1** Shared DE genes between CHIKV infected and non-infected SM/PM samples at 1 and 2 days post-feeding/infection (dpf/pi). **A** Venn diagram. **B** Description of shared DE genes. **Figure S2** Median logFC values for upregulated and downregulated DE genes in response to CHIKV infection and SM/PM feeding at 1 and 2 days post-feeding/post-infection (dpf/pi). logFC, logarithmic fold-change. **Figure S3** Median-normalized expression levels (at FPKM) of immunity related genes (**A**), RNAi pathway genes (**B**), and apoptotic pathway genes (**C**). Data show a comparison between sugarfed controls and SM/PM RNA-Seq libraries at 1 and 2 days post-feeding/post-infection (dpf/pi) and are presented as heatmaps for each transcript (vertical axis) at each time point (horizontal axis), with yellow and blue indicating high and low levels of expression, respectively. **Figure S4** Expression profiles of two putative serine collagenase genes in response to CHIKV infection and SM/PM/BM ingestion in midguts at 1, 2 and 4 days post-feeding/post-infection (dpf/pi). qRT-PCR was performed using total RNA extracted from midguts of mosquitoes, which had received a CHIKV containing or virus-free BM/PM/SM at 1, 2, and 4 dpf/pi. Midguts of sugarfed mosquitoes were used as control. Mean values with standard deviation (SD) from three independent experiments are shown. Significances between sugarfed and other samples were determined by Student *t* test (* at *P* ≤ 0.05, ** at *p* ≤ 0.01). *AeLT*, late trypsin, AAEL013284; *AeSP1*, putative serine collagenase 1 precursor, AAEL007432. (ZIP 14704 kb)

Additional file 3: DE genes in response to CHIKV infection in SM and PM samples. (XLSX 50 kb)

Additional file 4: GO of DE genes upregulated by CHIKV. (XLSX 33 kb)

Additional file 5: GO of DE genes downregulated by CHIKV. (XLSX 26 kb)

Additional file 6: Expression levels (at FPKM) of genes used to generate heatmaps for Additional file 2: Figure S3. (XLSX 28 kb)

Additional file 7: DE genes in response to SM/PM feeding compared to sugarfed control samples. (XLSX 1834 kb)

Additional file 8: GO of DE genes upregulated by SM and PM. (XLSX 830 kb)

Additional file 9: GO of DE genes downregulated by SM and PM. (XLSX 335 kb)

Additional file 10: Expression levels (at FPKM) of genes used to generate heatmaps for Fig. 6. (XLSX 24 kb)

Additional file 11: Primers used for qRT-PCR and recombinant protein expressions. (XLSX 9 kb)

## Abbreviations

ADAM(TS): A disintegrin and metalloproteinase (with thrombospondin motifs)
BL: Basal lamina
BM: Bloodmeal
CHIKV: Chikungunya virus
DE: Differentially expressed
dpf: Days post-feeding (no virus involved)
dpi: Days post-infection (with CHIKV)
FPKM: Fragments per kilobase of transcript per million mapped reads
GO: Gene ontology
LogFC: Logarithmic fold-change
MMP: Matrix metalloproteinase
PM: Protein meal
RT: Room temperature
SM: Saline meal
TIMP: Tissue inhibitor of metalloproteinases
