# Supplementary figures and images for: The midgut transcriptome of Aedes aegypti fed with saline or protein meals containing chikungunya virus reveals genes potentially involved in viral midgut escape
Source: BMC Genomics. 2017 May 15;18:382. doi: 10.1186/s12864-017-3775-6 (PMC5433025; doi:10.1186/s12864-017-3775-6)

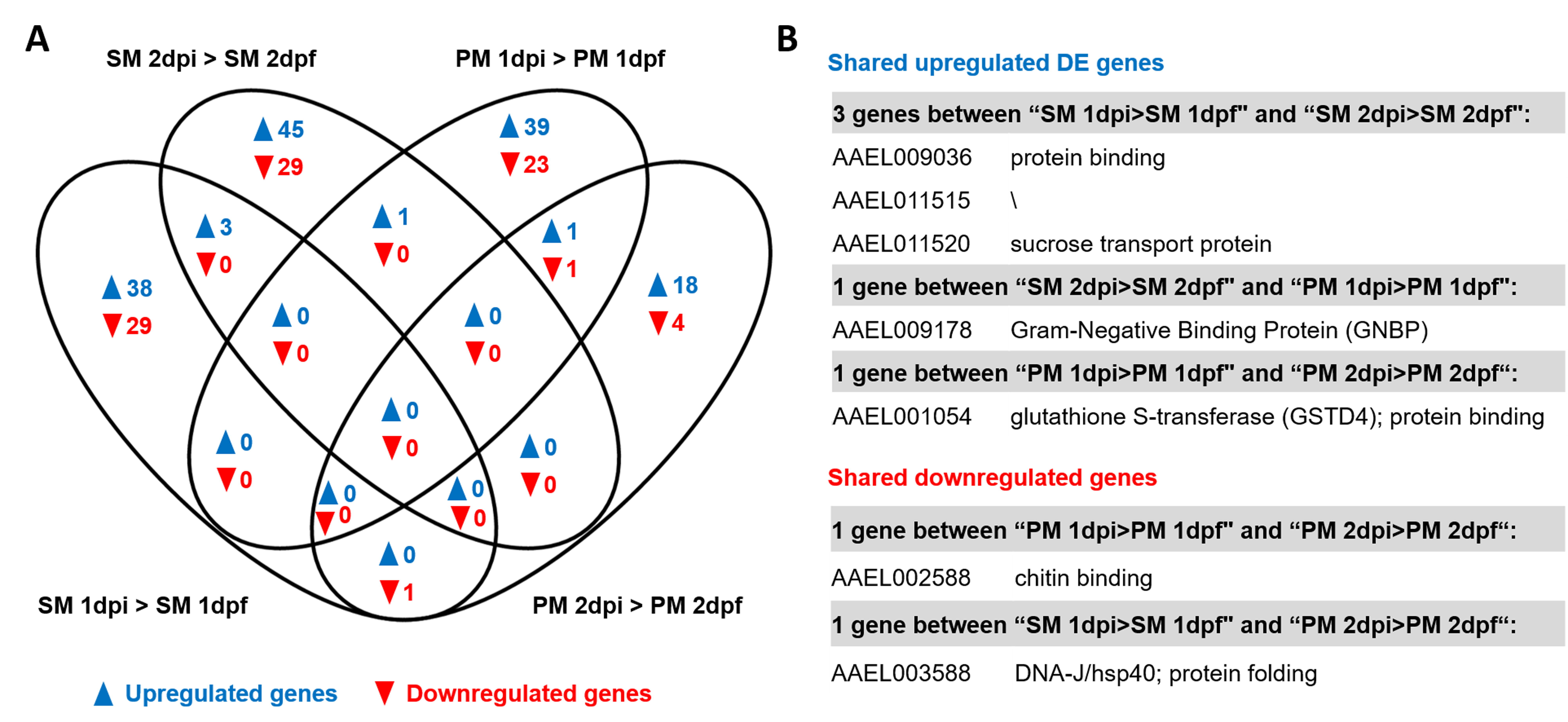

Supplement: Supplementary file 2 — Figure S1 Shared DE genes between CHIKV infected and non-infected SM/PM samples at 1 and 2 days post-feeding/infection (dpf/pi). A Venn diagram. B Description of shared DE genes. Figure S2 Median logFC values for upregulated and downregulated DE genes in response to CHIKV infection and SM/PM feeding at 1 and 2 days post-feeding/post-infection (dpf/pi). logFC, logarithmic fold-change. Figure S3 Median-normalized expression levels (at FPKM) of immunity related genes (A), RNAi pathway genes (B), and apoptotic pathway genes (C). Data show a comparison between sugarfed controls and SM/PM RNA-Seq libraries at 1 and 2 days post-feeding/post-infection (dpf/pi) and are presented as heatmaps for each transcript (vertical axis) at each time point (horizontal axis), with yellow and blue indicating high and low levels of expression, respectively. Figure S4 Expression profiles of two putative serine collagenase genes in response to CHIKV infection and SM/PM/BM ingestion in midguts at 1, 2 and 4 days post-feeding/post-infection (dpf/pi). qRT-PCR was performed using total RNA extracted from midguts of mosquitoes, which had received a CHIKV containing or virus-free BM/PM/SM at 1, 2, and 4 dpf/pi. Midguts of sugarfed mosquitoes were used as control. Mean values with standard deviation (SD) from three independent experiments are shown. Significances between sugarfed and other samples were determined by Student t test (* at P ≤ 0.05, ** at p ≤ 0.01). AeLT, late trypsin, AAEL013284; AeSP1, putative serine collagenase 1 precursor, AAEL007432. (ZIP 14704 kb) [file 12864_2017_3775_MOESM2_ESM.zip › Figure S1.tif]

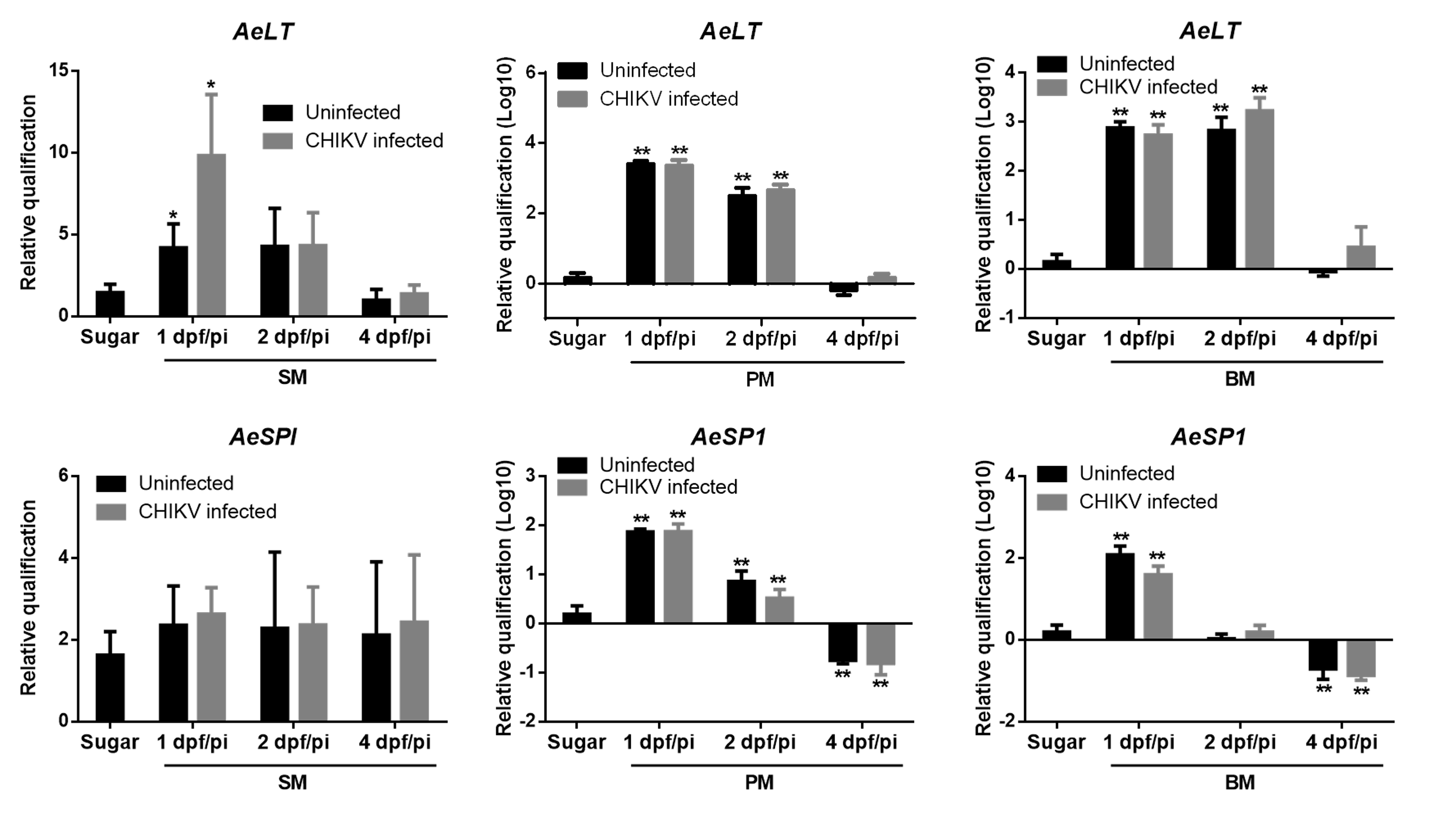

Supplement: Supplementary file 2 — Figure S1 Shared DE genes between CHIKV infected and non-infected SM/PM samples at 1 and 2 days post-feeding/infection (dpf/pi). A Venn diagram. B Description of shared DE genes. Figure S2 Median logFC values for upregulated and downregulated DE genes in response to CHIKV infection and SM/PM feeding at 1 and 2 days post-feeding/post-infection (dpf/pi). logFC, logarithmic fold-change. Figure S3 Median-normalized expression levels (at FPKM) of immunity related genes (A), RNAi pathway genes (B), and apoptotic pathway genes (C). Data show a comparison between sugarfed controls and SM/PM RNA-Seq libraries at 1 and 2 days post-feeding/post-infection (dpf/pi) and are presented as heatmaps for each transcript (vertical axis) at each time point (horizontal axis), with yellow and blue indicating high and low levels of expression, respectively. Figure S4 Expression profiles of two putative serine collagenase genes in response to CHIKV infection and SM/PM/BM ingestion in midguts at 1, 2 and 4 days post-feeding/post-infection (dpf/pi). qRT-PCR was performed using total RNA extracted from midguts of mosquitoes, which had received a CHIKV containing or virus-free BM/PM/SM at 1, 2, and 4 dpf/pi. Midguts of sugarfed mosquitoes were used as control. Mean values with standard deviation (SD) from three independent experiments are shown. Significances between sugarfed and other samples were determined by Student t test (* at P ≤ 0.05, ** at p ≤ 0.01). AeLT, late trypsin, AAEL013284; AeSP1, putative serine collagenase 1 precursor, AAEL007432. (ZIP 14704 kb) [file 12864_2017_3775_MOESM2_ESM.zip › Figure S4.tif]

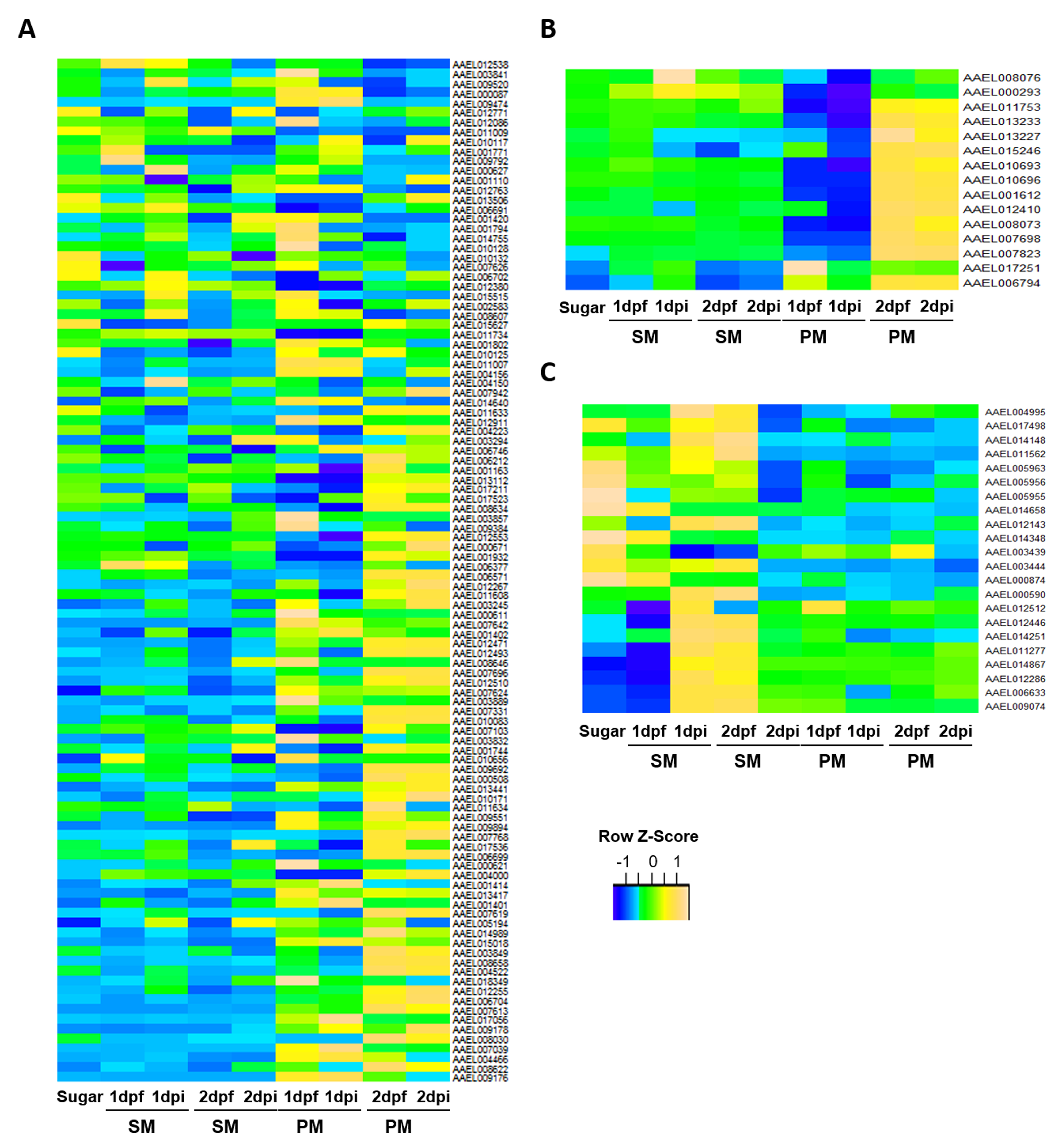

Supplement: Supplementary file 2 — Figure S1 Shared DE genes between CHIKV infected and non-infected SM/PM samples at 1 and 2 days post-feeding/infection (dpf/pi). A Venn diagram. B Description of shared DE genes. Figure S2 Median logFC values for upregulated and downregulated DE genes in response to CHIKV infection and SM/PM feeding at 1 and 2 days post-feeding/post-infection (dpf/pi). logFC, logarithmic fold-change. Figure S3 Median-normalized expression levels (at FPKM) of immunity related genes (A), RNAi pathway genes (B), and apoptotic pathway genes (C). Data show a comparison between sugarfed controls and SM/PM RNA-Seq libraries at 1 and 2 days post-feeding/post-infection (dpf/pi) and are presented as heatmaps for each transcript (vertical axis) at each time point (horizontal axis), with yellow and blue indicating high and low levels of expression, respectively. Figure S4 Expression profiles of two putative serine collagenase genes in response to CHIKV infection and SM/PM/BM ingestion in midguts at 1, 2 and 4 days post-feeding/post-infection (dpf/pi). qRT-PCR was performed using total RNA extracted from midguts of mosquitoes, which had received a CHIKV containing or virus-free BM/PM/SM at 1, 2, and 4 dpf/pi. Midguts of sugarfed mosquitoes were used as control. Mean values with standard deviation (SD) from three independent experiments are shown. Significances between sugarfed and other samples were determined by Student t test (* at P ≤ 0.05, ** at p ≤ 0.01). AeLT, late trypsin, AAEL013284; AeSP1, putative serine collagenase 1 precursor, AAEL007432. (ZIP 14704 kb) [file 12864_2017_3775_MOESM2_ESM.zip › Figure S3.tif]

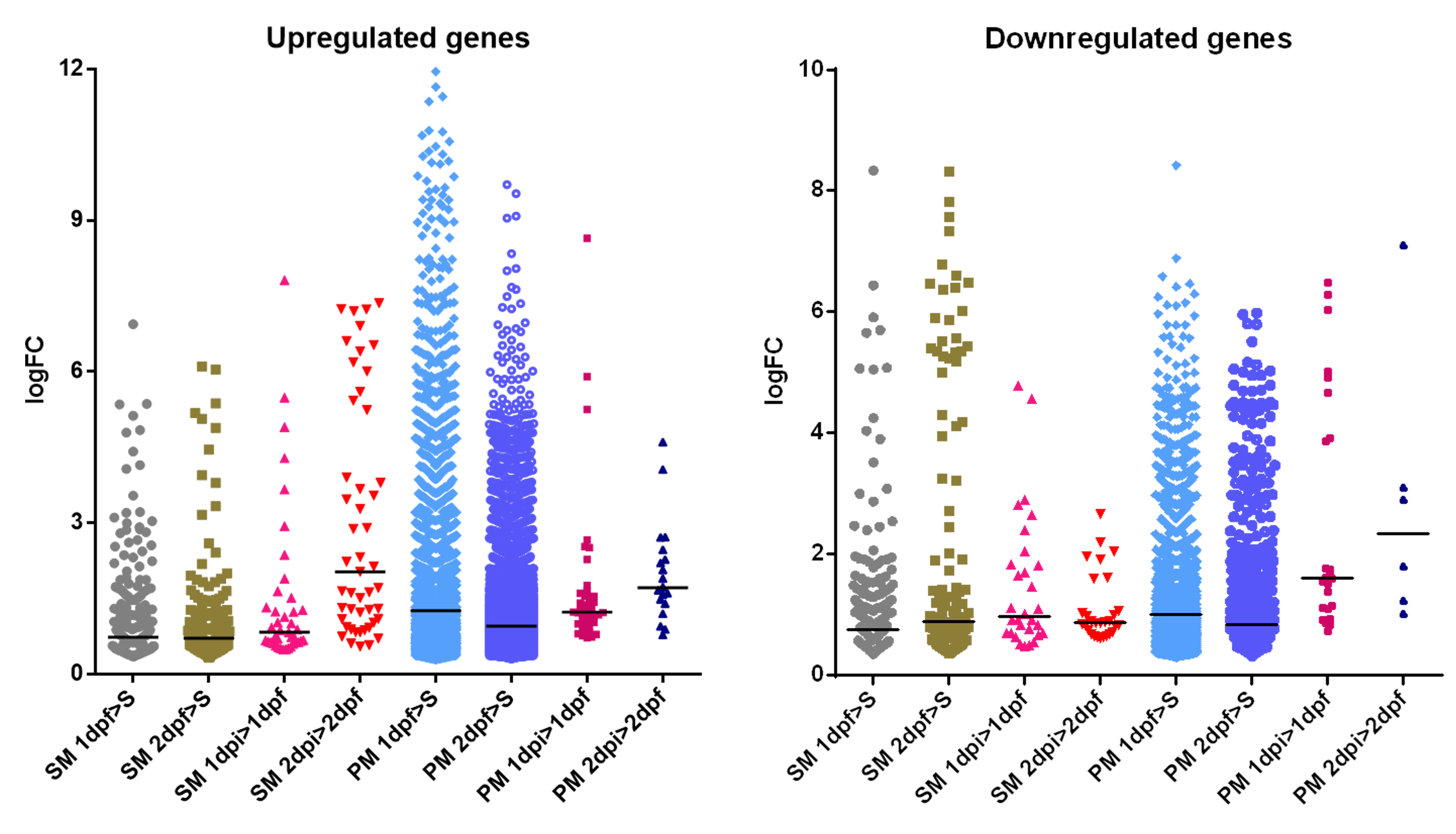

Supplement: Supplementary file 2 — Figure S1 Shared DE genes between CHIKV infected and non-infected SM/PM samples at 1 and 2 days post-feeding/infection (dpf/pi). A Venn diagram. B Description of shared DE genes. Figure S2 Median logFC values for upregulated and downregulated DE genes in response to CHIKV infection and SM/PM feeding at 1 and 2 days post-feeding/post-infection (dpf/pi). logFC, logarithmic fold-change. Figure S3 Median-normalized expression levels (at FPKM) of immunity related genes (A), RNAi pathway genes (B), and apoptotic pathway genes (C). Data show a comparison between sugarfed controls and SM/PM RNA-Seq libraries at 1 and 2 days post-feeding/post-infection (dpf/pi) and are presented as heatmaps for each transcript (vertical axis) at each time point (horizontal axis), with yellow and blue indicating high and low levels of expression, respectively. Figure S4 Expression profiles of two putative serine collagenase genes in response to CHIKV infection and SM/PM/BM ingestion in midguts at 1, 2 and 4 days post-feeding/post-infection (dpf/pi). qRT-PCR was performed using total RNA extracted from midguts of mosquitoes, which had received a CHIKV containing or virus-free BM/PM/SM at 1, 2, and 4 dpf/pi. Midguts of sugarfed mosquitoes were used as control. Mean values with standard deviation (SD) from three independent experiments are shown. Significances between sugarfed and other samples were determined by Student t test (* at P ≤ 0.05, ** at p ≤ 0.01). AeLT, late trypsin, AAEL013284; AeSP1, putative serine collagenase 1 precursor, AAEL007432. (ZIP 14704 kb) [file 12864_2017_3775_MOESM2_ESM.zip › Figure S2.tif]
